# Minocycline markedly reduces acute visceral nociception via inhibiting neuronal ERK phosphorylation

**DOI:** 10.1186/1744-8069-8-13

**Published:** 2012-02-24

**Authors:** Ik-Hyun Cho, Min Jung Lee, Minhee Jang, Nam Gil Gwak, Ka Yeon Lee, Hyuk-Sang Jung

**Affiliations:** 1 Department of Anatomy, College of Oriental Medicine, and Institute of Oriental Medicine, Kyung Hee University, Seoul 130-701, South Korea

**Keywords:** Minocycline, Acute visceral pain, c-Fos, p-ERK, Writhes

## Abstract

**Background:**

Minocycline prevents the development of neuropathic and inflammatory pain by inhibiting microglial activation and postsynaptic currents. But, how minocycline obviates acute visceral pain is unclear. The present study investigated whether minocycline had an any antinociceptive effect on acetic acid-induced acute abdominal pain after intraperitoneal (i.p.) administration of saline or minocycline 1 hour before acetic acid injection (1.0%, 250 μl, i.p.).

**Results:**

Minocycline (4, 10, or 40 mg/kg) significantly decreased acetic acid-induced nociception (0-60 minutes post-injection) and the enhancement in the number of c-Fos positive cells in the T5-L2 spinal cord induced by acetic acid injection. Also, the expression of spinal phosphorylated extracellular signal-regulated kinase (p-ERK) induced by acetic acid was reduced by minocycline pre-administration. Interestingly, intrathecal introduction of PD98059, an ERK upstream kinase inhibitor, markedly blocked the acetic acid-stimulated pain responses.

**Conclusions:**

These results demonstrate that minocycline effectively inhibits acetic acid-induced acute abdominal nociception via the inhibition of neuronal p-ERK expression in the spinal cord, and that minocycline may have therapeutic potential in suppressing acute abdominal pain.

## Background

Minocycline is a second-generation tetracycline antibiotic derivative that effectively crosses the blood-brain barrier [1], and which has a proven safety record in humans [2]. Minocycline has anti-inflammatory and neuroprotective effects in animal models of cerebral ischaemia [3,4], traumatic injury [5], glutamate-induced neurotoxicity [6], experimental autoimmune encephalonmyelitis [7], Huntington's disease [8] and Parkinson's disease [2,9,10]. Minocycline's efficacy has been bolstered by studies showing decreased secondary neuronal damage via the inhibition of microglial activation. Recently, it has been demonstrated that this minocycline-mediated microglial inhibition attenuates the development of pain hypersensitivity by inhibiting proinflammatory cytokine expression in rat models of both neuropathic pain and spinal immune activation by intrathecal human immunodeficiency virus-1 (HIV-1) gp120 [11,12]. Also, minocycline completely reverses mechanical hyperalgesia in diabetic rats through microglia-induced changes in the expression of the potassium chloride co-transporter 2 (KCC2) in the spinal cord [13]. In addition, we confirmed that minocycline attenuates tactile hypersensitivity following the trigeminal sensory nerve injury by inhibiting microglial p38 mitogen activated protein kinase (MAPK) activation [14], and that minocycline markedly reduced formalin-induced inflammatory pain by inhibition of excitatory postsynaptic currents (EPSCs) in the substantia gelatinosa [15]. These findings raise the possibility of the potential of minocycline as an analgesic for other types of pain models.

Visceral pain is the most common form of pain produced by disease, for which medical care is sought. Despite the conventional belief that visceral pain is a variant of somatic pain, it differs in neurological mechanisms and transmission pathways. Visceral pain is characterized by referral hyperalgesia and also it is not always linked to tissue injury [16,17]. Also, visceral pain that reflects the enhanced perception of physiological signals from the gut and/or the enhanced perception of experimental visceral stimuli along with hypervigilance to these aspects, is commonly considered to play a major role in the pathophysiology of irritable bowel syndrome (IBS) [17,18]. Various studies have evaluated the underlying mechanisms of visceral hypersensitivity and the influence of various stresses on the visceral pain pathways [17,18]. Recent studies have revealed the activation of ERK in spinal cord after noxious visceral stimulation [19,20].

ERK, a MAPK, could play a role in regulating nociceptive activities in primary sensory pathways after pathologic irritation of the peripheral system, such as peripheral nerve injury or inflammation [21-23]. Phosphorylation of ERK is observed in spinal dorsal horn neurons in response to noxious stimulation of the peripheral tissue, such as the injection of complete Freund's adjuvant (CFA) into a hindpaw [22], an intense noxious peripheral or C-fiber electrical stimulus [21], L5 spinal nerve ligation [23], and the injection of cyclophosphamide into the urinary bladder [24]. ERK was also shown to be phosphorylated in the spinal cord of a murine model of visceral pain and hyperalgesia, intracolonic instillation of either capsaicin or mustard oil [25], and in a model of acute inflammation and distention of the colon [26]. Intrathecal injection of specific inhibitor (U-0126 or PD-98059), which specifically attenuates ERK activity, can reduce nociceptive response behavior in the inflammatory pain, CFA-induced joint inflammation [27], and visceral pain by intracolonic capsacin [19]. These studies suggest an essential role of ERK in the development and maintenance of inflammatory or neuropathatic hyperalgesia. However, very little is known about the molecular signaling mechanisms evoked by acute visceral pain and there is no information on the involvement of ERK in the spinal processing in this type of pain.

The present study focused on the role of minocycline on spinal ERK in modulating acute visceral pain. The study hypothesis was that minocycline attenuates the acetic acid-induced visceral nociception by inhibiting the phosphorylation of neuronal ERK in the spinal cord.

## Results

### Minocycline inhibits acetic acid-induced abdominal contraction

**Figure 1:**
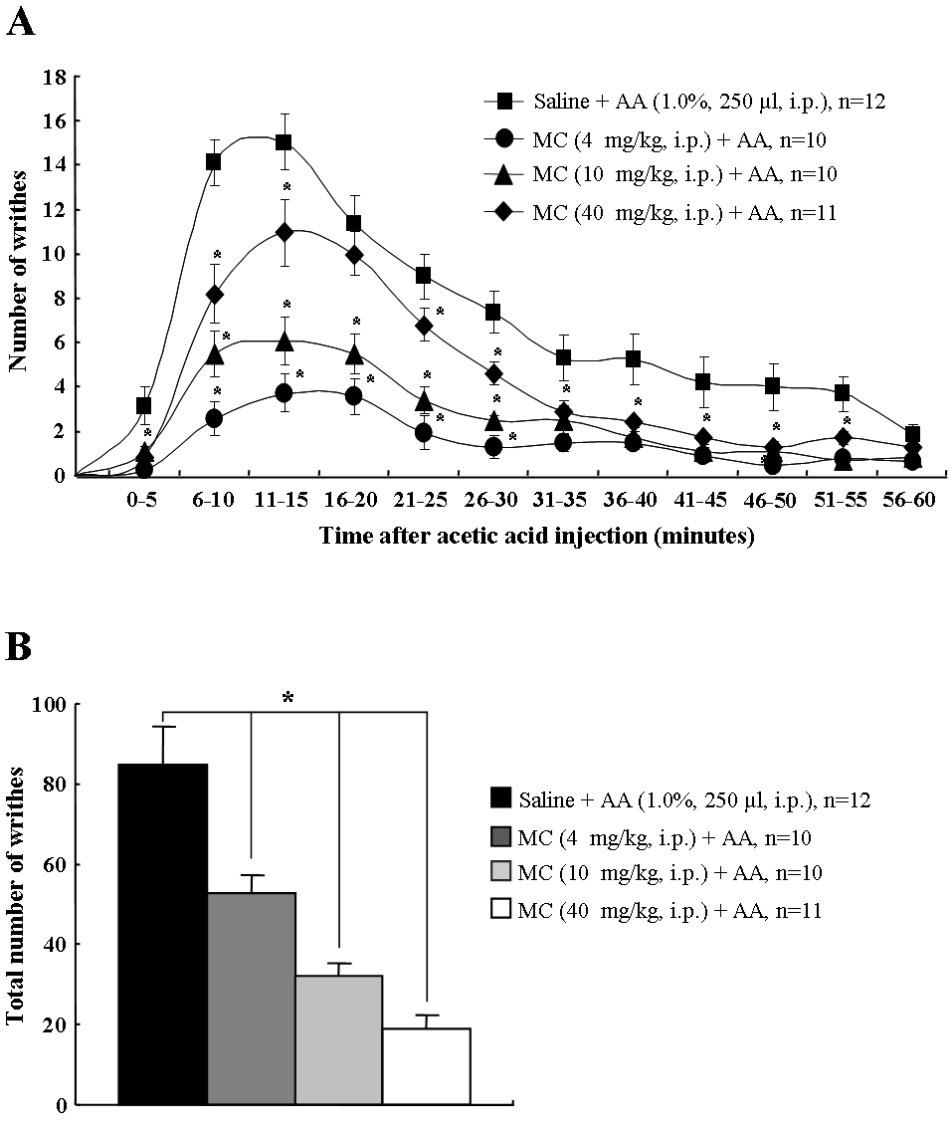
**Systemic administration of minocycline inhibits acetic acid-induced abdominal pain**. **(A) **Time course of 1% acetic acid (250 μl, i.p.)-induced visceral pain behavior (mean ± SE). Number of abdominal constrictions after acetic acid administration was significantly inhibited by pretreatment of minocycline in a dose dependent manner. **(B) **Total abdominal constrictions were attenuated by minocycline in a dose-related fashion following i.p. injection of acetic acid. Values are expressed mean ± SEM. *, P < 0.01 *versus *control group (saline-pretreated and acetic acid-treated animals (ANOVA test with a Fisher's post hoc test)

Acetic acid injection into the abdomen produces an acute visceral pain response [28]. In this study, for the 60 minutes following acetic acid administration (1.0%, 250 μl), the number of abdominal constrictions or writhes, such as lengthwise stretches of the torso with a concomitant concave arching of the back [28], were counted and totaled every 5 minutes. Saline-treated mice showed the typical abdominal stretching and constriction behaviors, which peaked at 10-15 minutes (15.1 ± 1.2), and then gradually declined. However, the peak pain responses by peritoneal irritation were significantly inhibited by pretreatment of minocycline in a dose dependent manner (4 mg/kg, 11.0 ± 1.5; 10 mg/kg, 6.1 ± 1.1; 40 mg/kg, 3.5 ± 0.8) (Figure 1A). The total number of writhing responses during the 60 minutes after acetic acid injection in the saline pretreatment group was 84.5 ± 8.7. However, the total number of writhes was significantly decreased by pretreatment of minocycline in a dose dependent pattern (4 mg/kg, 52.7 ± 4.6; 10 mg/kg, 32.0 ± 3.3; 40 mg/kg, 18.9 ± 3.5) (Figure 1B). Intraperitoneal (i.p.) injection of either saline or minocycline by themselves did not alter the behavior of the animals (data not shown). The results suggest that minocycline has an anti-nociceptive effect on acetic acid-induced acute visceral pain.

### Minocycline reduces acetic acid-induced c-Fos expression

**Figure 2:**
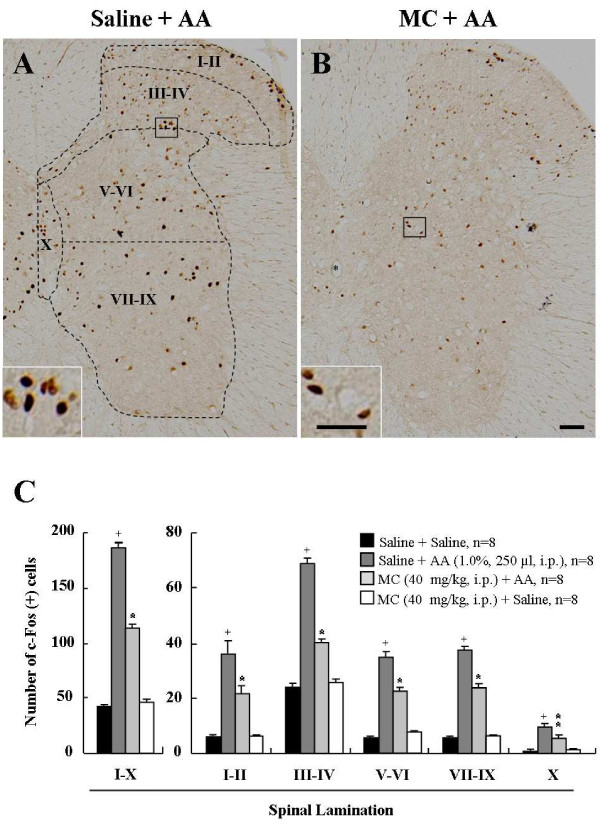
**Representative photomicrographs and graph showing immunoreactivity (IR) of c-Fos in the spinal cord (T5-L2)**. **(A, B) **c-Fos expression in the spinal cord of the saline/minocycline-pretreated and acetic acid-treated animals. Insets are high magnification of the open rectangles. The spinal gray matter was divided into the following four regions: (1) laminae I-II, (2) laminae III-IV, laminae V-VI, (4) laminae VII-IX, (5) laminae X. The scale bar denotes 100 μm. **(C) **The number of c-Fos positive cells in spinal cord following minocycline pre-injection. The enhancement in the number of c-Fos positive cells produced by acetic acid was significantly decreased in spinal cord by minocycline pre-administration. The mean number of c-Fos positive cells was calculated by averaging the total numbers of each region per each section/region. Values are expressed mean ± SEM. ^+^, *p *< 0.01 *versus *normal group; *, *p *< 0.01 and **, *p *< 0.05 *versus *control group (ANOVA test with a Fisher's post hoc test)

Because c-Fos, an immediate early gene protein product, is a neuroactive marker that can be used to analyze nociceptive pathways [25,29,30], we compared c-Fos expression between the saline- or acetic acid-treated mice (n = 8, in each group) after the time at which acetic acid maximally affected visceral pain (30 minutes after acetic acid injection) (Figure 1). c-Fosimmunoreactivity (IR) was evaluated in the T5-L2 spinal cord where primary afferents from splanchnic nerves innervate the entire gastrointestinal tract [17,31]. c-Fos-IR in the spinal cord was very scarce in normal mice (saline-administration alone) (I-X, 41.7 ± 2.2; I-II, 5.9 ± 0.5; III-IV, 24.0 ± 1.3; V-VI, 5.5 ± 0.6; VII-IX, 5.3 ± 0.6; X, 1.0 ± 0.1). The number of c-Fos positive cells in T5-L2 spinal cord was extensively increased by i.p. injection of acetic acid (I-X, 187.2 ± 5.0; I-II, 36.3 ± 1.9; III-IV, 69.2 ± 2.3; V-VI, 34.8 ± 1.3; VII-IX, 37.8 ± 1.7; X, 9.3 ± 0.5), but acetic acid-induced c-Fos-IR enhancement was significantly decreased by minocycline-pretreatment 1 hour prior to acetic acid administration (I-X, 114.2 ± 3.1; I-II, 21.6 ± 1.3; III-IV, 40.3 ± 1.3; V-VI, 22.8 ± 1.0; VII-IX, 24.1 ± 1.2; X, 5.4 ± 0.4). Minocycline, itself did not exert any effect on c-Fos expression in the spinal cord (Figure 2C). Taken together, the results suggest that minocycline has an inhibitory action for the excitation of spinal neurons in acetic acid-induced acute visceral pain. In addition, to investigate whether c-FOS was specifically activated in the T5-L2 spinal cord, we compared the expression of c-Fos in the samples from C1-C7, T5-L2 and L4-S1 spinal cord 30 minutes after acetic acid injection. c-Fos expression was upregulated in the T5-L2 levels, but not in the C1-C7, L4-S1, and normal T5-L2 segments (Additional file 1: Figure S1).

### Minocycline attenuates acetic acid-induced neuronal p-ERK expression

**Figure 3:**
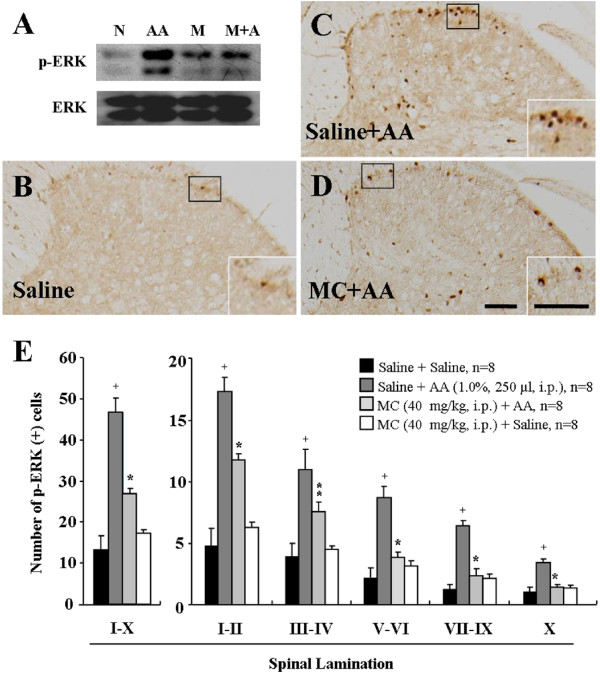
**Activation (phosphorylation) of spinal ERK1/2 after saline or minocycline pretreatment**. **(A) **Western blots of spinal tissue (T5-L2). N, normal. AA, saline-pretreated and acetic acid-treated mice, M, minocycline alone. M + A, minocycline-pretreated and acetic acid-treated mice. **(B-D) **Immunohistochemistry of spinal tissue from normal animals (B), saline-pretreated and acetic acid-treated mice (C), minocycline-pretreated and acetic acid-treated mice (D). Insets are high magnification of the open rectangles. The scale bar denotes 100 μm. **(E) **The number of p-ERK positive cells in spinal cord (T5-L2) following minocycline pre-injection. The enhancement in the number of p-ERK positive cells produced by acetic acid was significantly decreased in spinal cord by minocycline pre-administration. The mean number of p-ERK positive cells was calculated by averaging the total numbers of each region per each section/region. Values are expressed mean ± SEM. ^+^, *p *< 0.01 *versus *normal group; *, *p *< 0.01 and **, *p *< 0.05 *versus *control group (ANOVA test with a Fisher's post hoc test)

**Figure 4:**
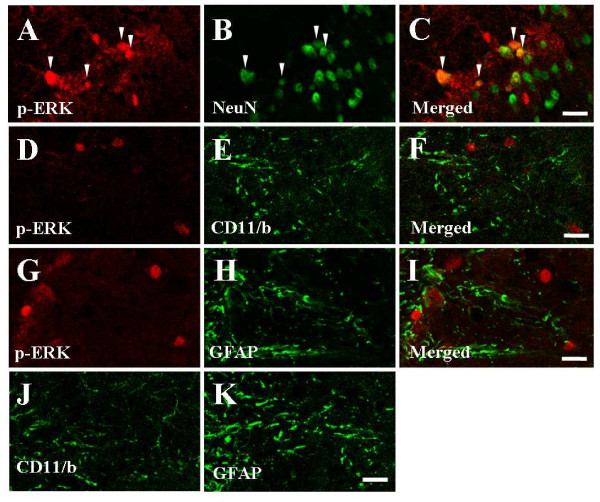
**Representative photomicrographs showing immunoreactivity (IR) of phospho-ERK (p-ERK) in the spinal dorsal horn (T12-L5)**. (A-I) p-ERK and NeuN immunoreactivity in the spinal dorsal horn of the saline-pretreated and acetic acid-treated mice. The p-ERK immunofluorescence was found exclusively in spinal neuron (C; merged image), but not in microglia (D, p-ERK; E, CD11/b; F, merged image) or astrocytes (G, p-ERK; H, GFAP; I, merged image). (J, K) Microglia (J, CD11/b) and astrocytes (K, GFAP) in normal mice. The scale bar denotes 50 μm

It has been demonstrated that spinal ERK is activated in experimental visceral pain models [19,26,27]. Therefore, we investigated whether minocycline could produce its effects through the spinal ERK pathway in the acetic acid-induced acute visceral pain. As shown in Figure 3A, 30 minutes after acetic acid administration, phosphorylation of ERK was clearly evident in the T5-L2 spinal cord. However, the elevated level of phospho (p)-ERK was decreased by minocycline-administration (Figure 3A). In addition, we examined the spinal distribution of p-ERK expression (Figures 3B-E). Immunohistochemical analysis indicated that p-ERK positive cells in the T5-L2 spinal cord were very scarce in saline-administrated mice (I-X, 13.2 ± 3.4) (Figures 3B, E). The number of p-ERK positive cells in the lamina I to X of the spinal cord was significantly increased by acetic acid-administration (I-X, 46.7 ± 3.4), but these acetic acid-stimulated p-ERK enhancement was significantly decreased by minocycline-pretreatment (I-X, 26.9 ± 1.3) (Figures 3C-E). To investigate the nature of the p-ERK positive cells, we examined whether ERK was activated in neuron, microglia, or astrocytes using multiple immunofluorescence method. Interestingly, the p-ERK immunofluorescence in the spinal cord from saline-treated mice was found exclusively in neuron (83.4%; 243 p-ERK IR and NeuN IR neurons/290 p-ERK IR neurons) (Figures 4AC), but was not clear in microglia or astrocytes (Figures 4D-I). Microglia and astrocytes were not sufficiently activated 30 minutes after acetic acid treatment (Figures 4E, H, J and 4K). These results suggest that minocycline attenuates neuronal ERK activation in the acetic acid-induced acute visceral pain. In addition, to investigate whether p-ERK is specifically increased in T5-L2 spinal segments, we compared the expression of p-ERK in the samples from C1-C7, T5-L2 and L4-S1 spinal segments 30 minutes after acetic acid injection. As expected, the phosphorylation of ERK was specifically increased in the T5-L2 levels, but not in the C1-C7, L4-S1, and normal T5-L2 spinal segments (Additional file 1: Figure S1).

### Microglia and astrocytes are not morphologically activated by acetic acid treatment

Because spinal glia (microglia and astrocytes) are activated in inflammatory pain [15,32,33] and neuropathic pain [12,34-36], and since minocycline attenuates behavioral hypersensitivity through the inhibition of microglial activation in these pain models [12,15,32], we examined whether spinal microglia and astrocytes were activated by acetic acid and, if so, whether the activated spinal microglia could be inhibited by minocycline pretreatment. Activated microglia usually display CD11/b or Iba-1 (a marker for microglia/macrophage lineage cells)-IR with enlarged cell body and much shorter and thicker processes [15,37]. However, when we analyzed CD11/b positive cells 30 minutes following acetic acid injection, microglial activation by acetic acid was not clearly found compared to normal mice (Figures 4E, J). In addition, we also confirmed that astrocytes similar to microglia were not enough activated 30 minutes following acetic acid injection, using glial fibrillary acidic protein (GFAP) antiserum (Figures 4H, K). The results indicate that the morphology of spinal microglia and astrocytes are not distinctly influenced by either acetic acid in acute visceral pain, and that spinal microglia and astrocytes do not directly contribute to acute visceral pain.

### Intrathecal administration of PD-98059 reduces acetic acid-induced abdominal constriction

**Figure 5:**
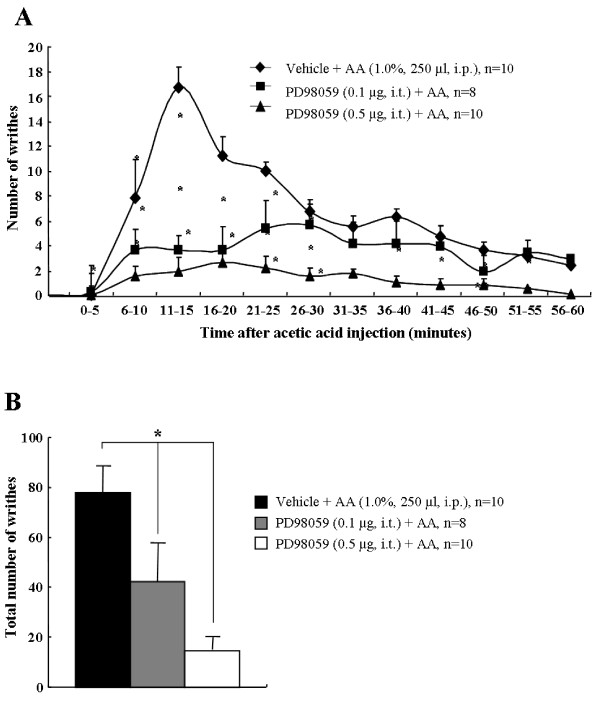
**Administration of PD-98059 inhibits acetic acid-induced abdominal pain**. **(A) **Time course of 1% acetic acid (250 μl, i.p.)-induced visceral pain behavior (mean ± SE). Number of abdominal constrictions (writhes) after acetic acid administration was significantly inhibited by intrathecal pretreatment of PD-98059 in a dose dependent manner. **(B) **Total abdominal constrictions were remarkably attenuated by intrathecal administration of PD-98059 in a dose-related fashion following i.p. injection of acetic acid. *, P < 0.01 *versus *control group (vehicle-pretreated and acetic acid-treated animals). (ANOVA test with a Fisher's post hoc test)

After acetic acid administration, abdominal pain response was increased and p-ERK expression was up-regulated mainly in spinal neurons, but not in glial cells, and the elevated pain response and p-ERK expression was reduced by pretreatment of minocyclin (Figures 1 and 4). This observation suggests that neuronal p-ERK expression contributes to acetic acid-induced abdominal pain. To address this issue, we directly introduced PD-98059, an ERK upstream kinase (MEK) inhibitor, to normal mice intrathecally 20 minutes before the injection of acetic acid. In the vehicle-treated mice, the number of acetic acid-induced writhes peaked at 10-15 minutes (16.7 ± 3.1) then gradually declined. The total number of writhes was 78.0 ± 10.5 for 60 minutes, similar to the result of Figure 1 (Figure 5A, B). However, these abdominal pain responses and total number of writhes were almost completely blocked by pretreatment of PD-98059 in a dose dependent manner at the peak time (0.1 μg, 5.6 ± 2.2;

0.5 μg, 2.6 ± 1.1) and total number of writhes (0.1 μg, 42.4 ± 15.6; 0.5 μg, 14.6 ± 5.2) (Figures 5A, B). These results indicate that intrathecal introduction of PD-98059 inhibits acetic acid-induced abdominal pain.

## Discussion

In this study, we investigated the potential of minocycline as an analgesic for acute visceral pain. Using the writhing test as an experimental model, we examined the effect of minocycline on acetic acid-induced pain response and spinal mechanism. The results indicate that (1) ERK activation is involved in nociceptive behavior and the excitation of spinal neurons in acetic acid-induced acute abdominal pain, (2) almost all cells with ERK activation are spinal neurons (T5-L2), (3) minocycline administrated 1 hour before acetic acid injection into the peritoneum markedly decreases c-Fos and p-ERK expression in the spinal cord (Figures 2 and 3), and (4) the intrathecal introduction of the MEK inhibitor, PD-98059, reduces acetic acid-induced abdominal pain (Figure 5). Collectively the data indicate that the ERK activation is involved in the pain behavior and neuronal response of acute abdominal pain, and that minocycline remarkably attenuates acute abdominal pain behavior and spinal c-Fos-IR, and that these effects might be a consequence of reduced spinal ERK activations.

As in other areas of pain research, the use of animal models is a necessary step to elucidate the underlying neurophysiological and neuropharmacological mechanisms of visceral pain. Over the past few years, a number of animal models have been developed that, to a large extent, mimic the nociception originating in the viscera [18,31]. These models have helped advance our understanding of the acute physiological responses associated with mechanical or inflammatory visceral nociception. It has become apparent that visceral pain and somatic pain are different, although some similarities exist [31]. In the present study, used the acetic acid-induced nociceptive model was developed for the screening of analgesic drugs and described as the writhing test [38] to exam the anti-nociceptive effect of minocycline in acute visceral pain. I.p. injection of acetic acid is a well known noxious chemical visceral stimulus in animals [38-44]. The nociceptive stimulation of the peritoneum by i.p. acetic acid produces abdominal contractions or writhes, and gastrointestinal ileus characterized by inhibition of gastric emptying and small intestine transit. Both procedures are associated with visceral pain [45-47]. This model is also used as a model of somatic-visceral pain [48,49].

In a recent study, systemically administered minocycline (15, 30, 45, or 100 mg/kg) reduced carrageenan- or formalin-induced paw edema, formalin-induced inflammatory nociceptive responses, and tactile hypersensitivity by following the sensory nerve injury [15,50]. In the present study, minocycline (4, 10, or 40 mg/kg), when intraperitoneally administrated 1 hour before acetic acid injection reduced writhing behavior in a dose dependent pattern (Figure 1). However, interestingly, a previous publication reported the contradictory results; a single dose of minocycline (30 or 100 mg/kg, i.p.) 30 minutes before acetic acid or zymosan injection did not attenuate the nociceptive behavior in mice. It had no effect on the early events of peritoneal inflammation (vascular permeability, inflammatory cell infiltration, and release of pro-inflammatory cytokines) in acetic acid or zymosan-injected mice. In addition, minocycline did not alter basal nociceptive responses in the tail immersion test [51]. They used male Swiss mice (20-26 g), and we used male ICR mice (20-25 g), they administrated minocycline 30 minutes prior to nociceptive stimuli, and we injected 1 hour before acetic acid stimulation, and they prepared minocycline by suspension them in one or two drops of Tween 80 in normal saline and we used normal saline. Furthermore, it has been reported that the concentration of minocycline in brain peaks at 1-2 hours after i.p. injection in phosphate buffered saline [52]. We suggest that differences in animal strain, preparation of drug, and duration for drug delivery could influence the nociceptive response.

c-Fos, an immediate-early gene protein product, is a neuroactive marker that can be used to indicate the activity of the spinal and supraspinal structures by many kind of peripheral pain including the visceral pain [49,53,54]. Previous studies showed that acetic acid enhanced central sensitivity with number of c-Fos-immunoreactive nuclei in the spinal cord [49,55]. Recent studies showed that ERK phosphorylation by noxious stimuli contributes to the neuronal c-Fos expression in the spinal cord. ERK phosphorylation is involved in the establishment as well as maintenance of long-term neuronal alterations associated with chronic pain following noxious stimulation, such as c-fiber/capsaicin stimulation [56], chronic constrictive injury-induced neuropathic pain [57], noxious bladder stimulation [58], and colorectal distension stimulation [26]. In this study, p-ERK expression was consistent with the high expression of c-fos in the laminae I-X of the spinal segments (T5-L2) which innervate gastrointestinal tract. These results suggest that ERK activation is correlated with the expression of spinal c-Fos in acetic acid-induced mice.

Phosphorylation of spinal ERK has a substantial role in nociception [22,59,60]. Recent studies have shown that ERK phosphorylation in the spinal dorsal horn neurons might also participate in some visceral pain processing by in a model of visceral pain and hyperalgesia induced by cororectal distension [61], bladder hyperalgesia by cyclophosphamide [20] and intracolonic instillation of irritants (capsaicin, mustard oil) in adult mice [19]. However, the role of spinal ERK in response to acetic acid-induced acute visceral pain has not been previously described. Our study clearly demonstrates that acetic acid can rapidly induce phosphorylation of ERK in the spinal cord (Figure 3). The p-ERK-IR neurons were specifically localized to T5-L2 segments, which is consistent with gastrointestinal innervations [17]. Therefore, we suggest that phosphorylation of spinal ERK is responsible for nociceptive transmission from the gastrointestinal tract stimulated by acetic acid.

Spinal microglia are activated in inflammatory and neuropathic pain [15,24]. Activated microglia secrete proinflammatory mediators such as prostaglandins, proteases, excitatory amino acids and cytokines such as tumor necrosis factor-alpha, interleukin (IL)-1β, and IL-6, and the released substances participate in pain processing [52]. Recently, it has been demonstrated that minocycline attenuates the development of pain hypersensitivity by the inhibition of microglial activation and proinflammatory cytokine expression in inflammatory and neuropathic pain [11,24]. Our previous studies confirmed that minocycline attenuates tactile hypersensitivity following trigeminal sensory nerve injury through the inhibition of microglial p38 MAPK activation [60], and that minocycline markedly reduces formalininduced inflammatory pain by inhibition of EPSCs in the substantia gelatinosa [15]. Recently, it was reported that minocycline completely reverses mechanical hyperalgesia in diabetic rats through microglia-induced changes in the expression of the potassium chloride co-transporter 2 (KCC2) at the spinal cord [13]. These findings raise the possibility that minocycline may behave as a potential analgesic for other types of pain model. Therefore, in the present study, we examined the anti-nociceptive effects of the minocycline in an acetic acid-induced acute visceral pain models. The results show that minocycline attenuates behavioral hypersensitivity through the inhibition of neuronal ERK activation in the spinal cord of acute visceral pain.

After peripheral nerve inflammation or injury, spinal microglia is initially activated and subsequently the sustained activation of astrocytes is precipitated. Activated astrocytes, as well as microglia, produce proinflammatory cytokines and chemokines, which are implicated in the induction and maintenance of pathological pain [62]. In our immunohistochemical study, the morphological activation of microglia and astrocytes were not clear 30 min after acetic acid injection, compared to normal brain (Figure 4). These findings agree with previous reports that the activation of microglia and astrocytes were not discernable as early as 1 h following formalin injection (acute pain) [15,33,63] and 2 h following the transection of the inferior alveolar nerve (chronic pain) [14], respectively. But in these reports, activated microglia and astrocytes were only increased after 1 day and peaked 3-7 days following formalin injection or peripheral nerve transection [14,15,33,63]. Therefore, our findings suggest that microglia and astrocytes might not be a causal factor in the central hypersensitivity of acetic acid-induced acute visceral pain.

MAPKs signalling pathways are likely important mechanism for development and maintenance of central sensitization [64,65]. The anti-nociceptive effect of an ERK upstream kinase (MEK) inhibitor, PD-98059, has been reported in various pain models including chronic constriction injury [66], complete Freund's adjuvant-induced monoartritis [67], and formalin-, capsaicin-, or mustard-induced inflammatory pain [19,21]. More recently, it was reported that increased spinal ERK1/2 phosphorylation in 3-week monosodium iodoacetate (MIA)-osteoarthritis (OA) rats was blocked by the PD-98059, when examined 30 minutes following acute intrathecal administration [68]. Moreover, the observations in MIA-OA rats that PD-98059 treatment partially blocks pain behaviour and reduces grip force strength [68] supports the potential involvement of ERK1/2 phosphorylation in the dorsal horn spinal cord in mediating nociceptive-induced central sensitization associated with this model of osteoarthritis. In the present study, because the acetic acid-stimulated acute abdominal pain was reduced by inhibiting neuronal p-ERK expression in spinal cord by pretreatment of minocycline (Figure 3), we studied whether the directly introduction of PD-98059 to subarachnoid space could reduce the acetic acid-induced acute abdominal pain. Consistent with previous findings, we confirmed that the intrathecal pre-administration of PD-98059 markedly blocks the excitation of spinal neuron in the mouse model of acetic acid-induced acute abdominal pain.

It is interesting to note that minocycline inhibits the early activation of neuronal ERK MAPK in spinal cord although the effect of minocycline is generally considered to be on glia. Based on the early/acute response of the visceral pain, the acute inhibitory effect of minocycline on ERK activation might not be due to its inhibitory actions on spinal glial activation. Minocycline might have a direct inhibitory effect on neuronal ERK rather than glial ERK. Thus, these results suggest that the anti-nociceptive effect of minocycline for acute visceral pain is associated with neuronal ERK activation in the spinal cord.

## Conclusions

Increased spinal ERK phosphorylation is important for pain behaviors based on the MEK inhibitor studies. However, the direct link between minocycline's inhibitory effects in visceral nociceptive responses and its modulating effects on p-ERK expression has been unclear. In this study, minocycline attenuated the abdominal nociception, and the activation of spinal c-Fos and ERK 1/2 in the acetic acid-induced acute visceral pain. The intrathecal introduction of PD-98059, a MEK inhibitor, reduced the nociceptive behavior by acetic acid. These results strongly suggest that minocycline has an anti-nociceptive effect on acetic acid-induced acute visceral pain by inhibiting neuronal ERK activation in the spinal cord.

## Methods

### Animals

The male ICR mice (weight, 20-25 g) were kept at a constant temperature of 23 ± 2°C with a 12-h light-dark cycle (light on 08:00 to 20:00), and fed food and water *ad libitum*. The animals were allowed to habituate to the housing facilities for 1 week before the experiments. All experiments were approved by the Institutional Animal Care and Use Committee (IACUC) in College of Oriental Medicine, Kyung Hee University and animal treatments were performed according to the guidelines of the International Association for the Study of Pain [69].

### Acetic acid-induced writhing test

The acetic acid-induced visceral pain model is widely used in experimental research to produce abdominal contractions [38-44]. Mice were randomly assigned to two groups, saline-treated group (control group, n = 12) and minocycline-treated group (n = 31). Minocyclinetreated mice received minocycline (Sigma-Aldrich, U.S.A.) either 4 mg/kg (n = 10), 10 mg/kg (n = 10) or 40 mg/kg (n = 11) intraperitonially (i.p.) 1 hour before acetic acid i.p. injection (1.0%, 250 μl), respectively. Control mice received an equal volume of saline vehicle. The dosage of minocycline was determined based on the previous reports of therapeutic effects of minocycline [3,4,10,12,15]. The time point of acetic acid injection was determined based on a previous report of optimal delivery of minocycline in rats [70]. Following the i.p. injection of acetic acid, mice were placed in a clear plastic cage (20 × 26 × 12 cm), and the number of writhes per mice was counted in 5 minutes interval for 60 minutes. The behavioral tests were performed blinded under the constant condition (temperature, 23 ± 2°C; humidity, 55 ± 5%) between 9:00 am and 12:00 am in a quiet room.

### Immunocytochemical evaluation

At 30 minutes after the i.p. injection of acetic acid, the mice used for immunohistochemistry (n = 8/group) were anesthetized with 40 mg/kg sodium pentobarbital (i.p.), and perfused with fresh 4% paraformaldehyde in 0.1 M phosphate buffer (pH 7.4). The T5-L2 spinal segment was removed and postfixed at 4°C overnight and then cryoprotected in 0.1 M PBS (pH 7.4) containing 30% sucrose for 48 hours at 4°C. Immunostaining was carried out according to previously established procedures [15,37]. Briefly, cryosections (10 μm thickness) were mounted onto gelatin-coated slide glass. Six transverse sections in 700 μm intervals were selected from each animal [saline, n = 8; minocycline (40 mg/kg), n = 8] and incubated for 30 minutes with 3% H_2_O_2_, and then blocked with a solution containing 5% normal goat/or horse serum, 2% BSA, 2% FBS and 0.1% triton X-100 for 2 hours at room temperature (RT). The sections were incubated overnight at 4°C with either rabbit anti-c-Fos (1:10,000; Oncogene, U.S.A.), or rabbit anti-phospho ERK (1:500; Cell Signaling, U.S.A.). Sections were then incubated with biotinylated rabbit IgG antibody (1:200; Vector Laboratories, U.S.A) for 1 hour at RT. After rinsing, the sections were incubated with avidin-biotinylated HRP complex (1:200; Vector Laboratories, U.S.A) for 1 hour at RT and visualized with DAB. Sections were rinsed, and dehydrated and cover-slipped. Immunostained images were captured using image system (DP 70 digital camera, Olympus, Japan) under light microscope. Photo-images from the right side of T5-L2 were drawn using manual technique, and the numbers of c-Fos positive cells were counted. Each section was divided into (1) superficial dorsal horn (laminae I-II), (2) deep dorsal horn (laminae III-IV), (3) neck region (laminae V-VI), (4) ventral horn (laminae VII-IX), and (5) central canal region (lamina X) [71]. Evaluation of the immunostained sections were performed by an experimenter unaware of the experimental condition.

### Immunofluorescence evaluation

For double immunofluorescent staining, sections were incubated overnight at 4°C with a mixture of rabbit anti-p-ERK antibody (1:500; Cell Signaling, U.S.A.) and mouse anti-NeuN (1:500; Chemicon, U.S.A)/or rat/mouse anti-CD11/b (1:200; Serotec, U.S.A.)/GFAP (1:1,000; Chemicon, U.S.A) antibody. The sections were then incubated for 1 hour at RT with mixture of Cy3- and FITC-conjugated rabbit/rat/mouse IgG antibody (1:200; Jackson ImmunoResearch, U.S.A.), and then examined with confocal imaging system (LSM 5 PASCAL; Carl Zeiss, Germany).

### Western blot analysis

To investigate the level of p-ERK expression, minocycline (40 mg/kg) was injected intraperitoneally 1 hour before the i.p. injection of acetic acid. And at 30 minutes after the i.p. injection of acetic acid, the mice used for Western blot analysis (n = 4/group) were anesthetized, and the C1-C7, T5-L2, and L4-S1 spinal segments were removed with lysis buffer (50 mM Tris-Cl, pH 7.5, 150 mM NaCl, 1% Triton X-100, 10% glycerol, and protease inhibitor mixture). A total of 50 μg of tissue lysate from each sample was resolved by electrophoresis on a 10% SDS-PAGE. The proteins were then transferred to PVDF membranes and blocked with 5% nonfat dry milk in Tween 20-containing Tris-buffered saline (TBST, 20 mM Tris, pH 7.4, 0.1% Tween 20, and 150 mM NaCl). The membranes were probed overnight with rabbit anti-c-Fos (1:1,000; Merck KGaA, Germany) or rabbit anti-p-ERK (1:2,000; Cell Signaling, U.S.A.) antibodies at 4°C, which was followed by incubation with HRP-conjugated secondary antibody at RT for 1 hour prior to enhanced chemiluminescence ECL (Amersham Pharmacia Biotech, U.S.A.) treatment and exposure to x-ray film. For normalization of antibody signal, the membranes were stripped and reprobed with antibodies for actin (1:2,000; Santa Cruz, U.S.A.) or ERK 1/2 (1:2,000; Cell Signaling, U.S.A.). After Western blot was performed several times, the density of each band was converted to numerical values using an Adobe Photoshop CS2 program (Adobe, San Jose, CA, U.S.A.), subtracting background values from an area of film immediately adjacent to the stained band. Data are expressed as the ratio of c-Fos or p-ERK against actin or total ERK 1/2 for each sample.

### Intrathecal (i.t.) administration of PD-98059

The i.t. injections were performed under light isofluran anesthesia (1-2%). The dorsal fur of each mouse (saline + acetic acid, n = 10; 0.1 μg PD-98059 + acetic acid, n = 8; 0.5 μg PD98059 + acetic acid, n = 10) was shaved, the spinal column was arched, and a 30-gauge needle was directly inserted into the subarachnoid space, between the L5 and L6 vertebrae [72]. Correct i.t. positioning of the needle tip was confirmed by manifestation of a characteristic tail flick response. The 0.1 and 0.5 μg of PD-98059 (2-amino-3'-methoxyflavone; Calbiochem, USA), an ERK upstream kinase (MEK) inhibitor, or vehicle (saline alone or 10% DMSO) were slowly injected into the mice with a 50 μl Hamilton micro syringe in a total volume of 5 μl. The entire injection procedure, from the induction of anesthesia until recovery of consciousness, took 4-5 minutes. Preliminary injections were performed with a similar volume of 10% India ink solution, and then the reliability and accuracy of this method was confirmed by subsequent dissection of the lumbar spinal cord. The success rate for the prior injections with this technique was over 97.5%. The same investigator performed all injections. The i.p. injections of acetic acid and behavioral test were performed 20 minutes after i.t. injection of PD-98059, as described above.

### Statistical analysis

The statistical significance of differences between the values was determined using an ANOVA with a Fisher's post hoc test. All data are presented as the mean ± S.E. and a statistical difference was accepted at the 5% level unless indicated otherwise.

## Competing interests

The authors declare that they have no competing interests.

## Authors' contributions

IHC conceived all experiments, performed immunohistochemistry, analyzed the results and prepared figures, and wrote the manuscript. MJL performed behavioral experiments and Western blot. MJ, NGG and KYL assisted with behavioral experiments. HSJ participated in the design of the study and drafted the manuscript with IHC. All authors have read and approved the final manuscript.

## Supplementary Material

Additional file 1 **Figure S1**. Expression of c-Fos and p-ERK in the spinal cord of acetic acid-induced visceral pain models. c-Fos (A, B) and p-ERK (C, D) are specifically upregulated in the T5-L2 spinal segments of acetic acid-treated mice. Values, expressed as relative intensities, represent the mean ± SEM. *P < 0.01 *versus *five control groups except acetic acid-treated T5-L2 segments (ANOVA test with a Fisher's post hoc test). S, saline-treated normal mice; AA, acetic acid-treated mice. Click here for file
