# Supplementary figures and images for: Minocycline markedly reduces acute visceral nociception via inhibiting neuronal ERK phosphorylation
Source: Mol Pain. 2012 Feb 24;8:13. doi: 10.1186/1744-8069-8-13 (PMC3342906; doi:10.1186/1744-8069-8-13)

# Supplementary Figure 1

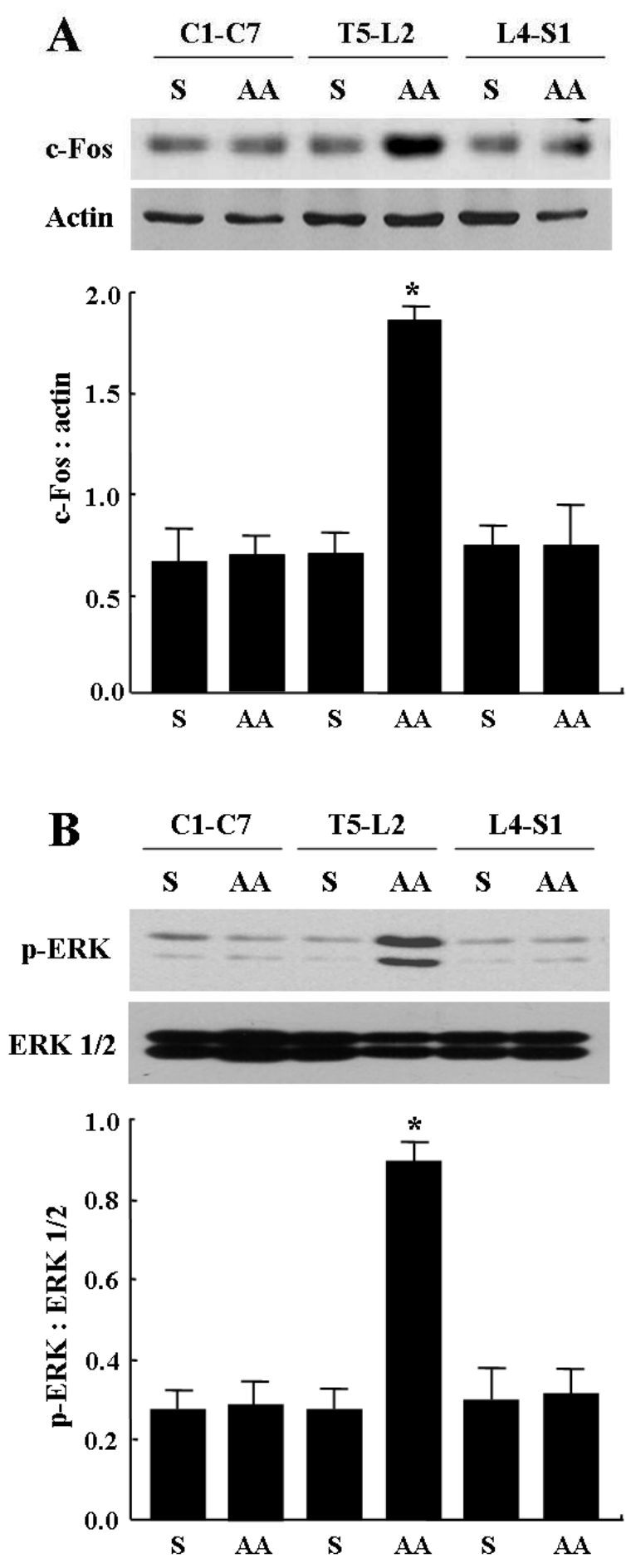

Supplement: Additional file 1 — Figure S1. Expression of c-Fos and p-ERK in the spinal cord of acetic acid-induced visceral pain models. c-Fos (A, B) and p-ERK (C, D) are specifically upregulated in the T5-L2 spinal segments of acetic acid-treated mice. Values, expressed as relative intensities, represent the mean ± SEM. *P < 0.01 versus five control groups except acetic acid-treated T5-L2 segments (ANOVA test with a Fisher's post hoc test). S, saline-treated normal mice; AA, acetic acid-treated mice. [file 1744-8069-8-13-S1.PDF]
